# Psychometric evaluation of the self-undermining scale in South Africa using the Rasch model

**DOI:** 10.4102/ajopa.v7i0.163

**Published:** 2025-05-20

**Authors:** Sergio L. Peral

**Affiliations:** 1 Department of Psychology, Faculty of Humanities, University of the Witwatersrand, Johannesburg, South Africa

**Keywords:** self-undermining scale, Rasch analysis, psychometric properties, reliability, validity, South Africa

## Abstract

The self-undermining scale is used to assess employee behaviours that undermine job performance, including making mistakes, creating conflict, creating confusion and creating a backlog in work tasks. To date, its psychometric properties have not been thoroughly investigated using item-response theory applications, especially in South Africa. Applying the Rasch Rating Scale Model, this study aimed to investigate the reliability and internal validity of the self-undermining scale, including item fit and rating scale functionality. Data were collected from 318 South African employees using a non-experimental, cross-sectional survey design. The instrument demonstrated unidimensional scaling with Cronbach’s alpha and person separation values of 0.77 and 1.57, respectively. Item and category fit statistics showed satisfactory fit to the Rasch model, with only one item warranting further attention. Some refinements regarding item wording and rating scale optimisation are provided.

**Contribution:**

This study is the first to investigate the reliability and validity of the self-undermining scale through the Rasch Measurement Model. It also offers cautionary insights into the applicability of the scale to measure self-undermining among South African employees because of the lack of discriminatory power. Recommendations for further validation studies are provided.

## Introduction

The self-undermining scale (Bakker & Wang, 2020) is a six-item self-report assessment designed to measure the extent to which employees engage in detrimental work behaviours such as making mistakes, creating confusion and conflict, poor communication and creating a backlog in work tasks (Bakker, 2015; Bakker & Costa, 2014). These concrete work behaviours lead to increased distractions and work pressure, and in turn, undermine employee well-being and performance (Roczniewska & Bakker, 2021).

Bakker and Wang (2020) developed and validated the self-undermining scale in global regions spanning Asia, the Americas and Europe. They used classical test theory (CTT), such as exploratory and confirmatory factor analysis, to evaluate the reliability and validity of the instrument. Although initial findings showed promise for the scale’s psychometric properties, it was noticed that some items functioned differently. Available information points to some variability in the factor loadings across countries and cultures (Bakker & Wang, 2020). Given the increased research interest on self-undermining (SU) behaviours internationally (Bakker & Costa, 2014; Bakker & De Vries, 2021; Bakker & Wang, 2020; Bakker et al., 2023) and in South Africa (Peral, 2019; Theron, 2022), it is a good time to investigate the psychometric properties of the scale in the context where it is used to ensure generalisability across contexts.

The aim of this research is to investigate the internal validity and reliability of the self-undermining scale within the South African context. Specifically, the Rasch Rating Scale Model is applied to evaluate the dimensionality of the scale, the difficulty of the items and how well the rating scale distinguishes between low and high self-underminers. Although Bakker and Wang’s (2020) use of CTT has credit, applications of the Rasch Measurement Model offer unique advantages. For example, the Rasch model enables researchers to determine the difficulty of the items and shows precisely where an item is measuring the underlying construct of interest (Bond & Fox, 2007; Hambleton & Jones, 1993). Using Rasch analysis, it will be possible to determine if the items comprising the self-undermining scale effectively measure individuals’ SU behaviour and for which persons the scale is most reliable.

Self-undermining is an emerging concept in the organisational behaviour literature and limited knowledge exists about the SU behaviours in which employees engage in (Bakker & Wang, 2020). When employees are overly stressed, their ability to self-regulate – think, feel and behave effectively – diminishes and leads them to creating obstacles that hinder their job performance (Roczniewska & Bakker, 2021). Self-undermining has been conceptualised within the nomological network of the job demands-resources (JD-R) theory, which classifies jobs into job demands and job resources (Bakker & Demerouti, 2017). Job demands (e.g. workload, conflict, pressure) are those aspects of the job requiring sustained physical, cognitive and/or emotional efforts, and are associated with psychophysiological strain (Demerouti et al., 2001). Contrastingly, job resources are those work-related aspects that help employees achieve work-related goals, stimulate their learning and growth potential, and buffer the negative impact of job demands (Demerouti et al., 2001).

Empirical evidence in various cultural contexts has demonstrated the negative implications of SU. In China, Bakker and Wang (2020) found SU to be positively related to burnout and negatively related to supervisor ratings of job performance. Similar findings were observed by Bakker et al. (2023) in a weekly-diary study among Dutch employees, where weekly demands positively related to SU through weekly burnout symptoms. In another daily dairy study, Polish nurses with high levels of burnout were more likely to engage in SU behaviour (Roczniewska & Bakker, 2021). Within the South African context, Peral (2019) found that SU mediated the relationship between the dark triad of personality and job performance. Specifically, employees scoring high on psychopathy were more likely to self-undermine, and in turn, experience decreased levels of job performance. Lastly, Theron (2022) investigated the impact of SU on employee engagement and burnout but yielded non-significant findings. Overall, these international and local findings suggest that acts of SU result in an unhealthy loss cycle of excessive job demands, job strain, burnout and lower work performance.

## The self-undermining scale

A two-wave study was initiated to develop and validate the self-undermining scale (Bakker & Wang, 2020). In the first study, a set of 10 items were translated from Dutch into English, Romanian, Spanish and Chinese. After expert evaluation, the 10-item scale was piloted to a sample of Chinese employees (*n* = 303) to determine the scale’s factorial validity and reliability using exploratory factor analysis (EFA). A one-factor solution explaining 55% of the variance and high factor loadings (> 0.62) for all items was found, with a Cronbach’s alpha of 0.92. Four items were removed as they were found to be more related to attribution and coping styles rather than actual behaviours (Bakker & Wang, 2020). A new EFA with the remaining six items again supported the one-factor solution, explaining 54% of the variance with high factor loadings for all items (> 0.66) and a Cronbach’s alpha of 0.88. A multi-group confirmatory factor analysis (MCFA) was then conducted on the samples from the United States (US), Chile, Romania and the Netherlands. The results indicated that an unconstrained one-factor model fit the data best, with a mean factor loading of 0.61 across the four countries. However, the MCFA showed that one-factor loading (item not mentioned by authors) differed in Chile (0.33) and the Netherlands (0.35), indicating variations in interpretation. Acceptable reliability for the scale was found across the countries, with reliability coefficients ranging from 0.70 (Chile) to 0.87 (US) across the samples.

The criterion-related validity of the self-undermining scale was tested in the second study. Bakker and Wang (2020) administered the instrument to two Chinese samples (*n* = 297, *n* = 298) and found that scores on SU were positively related to self-handicapping, supervisor-reports of SU and burnout; while negative correlations were reported between SU, personal initiative and work engagement, providing evidence for the scale’s convergent, predictive and discriminant validity. While preliminary findings lend support to the validity and reliability of the self-undermining scale, there is more to be known about its psychometric properties. Specifically, how well the items measure SU behaviour across the latent trait, how easy or difficult the items are to endorse and how well the rating scale distinguishes between low and high self-underminers remain as questions to be answered.

## Rasch measurement model

The Rasch model (Rasch, 1960), often classified under the umbrella of item response theory (IRT) models (Boone et al., 2014), is a latent trait psychometric model that provides a theoretical measurement framework to determine if measurement quality and precision has been achieved (Bond & Fox, 2007). According to the Rasch model, a person’s response to any given item is a function of a person’s ability and the difficulty of the item – this is often expressed graphically along a Wright map (Wright, 1984). A Wright map is a person-item map showing the position of persons’ abilities and item difficulties along the latent trait on the same metric, making it possible to determine how well the items measure the attribute of each person being measured.

Similar to traditional fit statistics used to determine model fit in CTT, the concept of *fit* carries significant weight in Rasch modelling. The fit of the items to the Rasch model is assessed by inspecting a set of fit statistics, namely infit and outfit mean square statistics. Values greater or less than one for these statistics indicate misfit and detract from quality measurement (Linacre, 2016).

Various iterations of the Rasch model are applied depending on the type of data, such as dichotomous or polytomous data. As the self-undermining scale uses polytomous data, the Andrich Rating Scale Model was employed in this study because it constrains the category thresholds to be equal across all items, making it more restrictive in nature (Bond & Fox, 2007). Furthermore, Fox and Jones (1998) assert that the Rating Scale Model is ideal for polytomous data with a consistent response format across all items, as is the case for the self-undermining scale items. One advantage is that fewer parameters are estimated, which can lead to better precision when estimating item thresholds and difficulties.

## Methods

### Participants

Non-probability convenience sampling was used to obtain a sample of 318 South African working adults. The sample consisted of more self-identified women (*n* = 182, 57.23%) than self-identified men (*n* = 136, 42.77%). The mean age of the participants was 35.52 (standard deviation [s.d.] = 11.29) with an average organisational tenure of 6 years. The ethnicity of the participants was as follows: White (*n* = 235, 73.89%), black African (*n* = 50, 15.72%), Indian (*n* = 16, 5.03%), mixed race (*n* = 16, 5.03%) and ‘Other’ (*n* = 1, 0.31%). In terms of the home languages spoken by the participants, majority were English speaking (*n* = 225, 70.75%), followed by Afrikaans (*n* = 41, 12.89%), isiZulu (*n* = 13, 4.08%) and Xitsonga (*n* = 10, 3.14%). The remainder of the sample indicated that they spoke other local South African languages (e.g. Siswati, Sesotho, Sepedi). Majority of the sample were employed at the time of data collection (*n* = 286, 89.93%), with 21 participants (6.60%) being self-employed, 7 (2.20%) being part-time employees and 4 (1.25%) choosing ‘Other’. Participants worked in various industries including education, banking, information technology, marketing and business consulting. The education profile of the sample was as follows: Undergraduate degree (*n* = 68, 21.38%), Diploma (*n* = 66, 20.75%), Grade 12 certificate (*n* = 50, 15.72%) and post-graduate (*n* = 121, 38.05%).

### Procedure

An online survey containing an information sheet, informed consent form and the self-undermining scale was created on Google Forms. A link to the survey was distributed via email and various social networking platforms (i.e. LinkedIn, Facebook, WhatsApp) to encourage participation. Emails and announcements listed the criteria for participation, which included (1) being a South African employee over the age of 18 and (2) could read and understand English. Participants were encouraged to share the survey link with their colleagues and invite other interested and willing individuals to take part in the study. Upon accessing the link, participants were presented with an introduction to the questionnaire, detailing the study’s purpose and emphasising its anonymous, confidential and voluntary nature. Follow-up emails and online posts were periodically sent as reminders to encourage participation.

### Instruments

The self-undermining scale (Bakker & Wang, 2020) was used to measure the SU behaviours of the South African working sample. The unidimensional scale consists of six items rated on a 5-point Likert type scale (1 = Never, 2 = Sometimes, 3 = Regularly, 4 = Often, 5 = Very Often). Example items include ‘I make mistakes’ and ‘I run into problems at work’. In their weekly-diary study, Bakker and De Vries (2021) reported alpha coefficients ranging from 0.84 to 0.88, while Roczniewska and Bakker (2021) reported an Omega (ω) reliability coefficient of 0.60 and 0.80 for the day and person level, respectively. Finally, Theron (2022) administered the self-undermining scale to academic staff in South Africa and found acceptable internal consistency (*a* = 0.71).

### Data analysis

The analyses were conducted using Winsteps (*version 4.4.8;* Linacre, 2019) using joint maximum likelihood estimation. Dimensionality was investigated through a principal components analysis (PCA) of the standardised residuals (Linacre, 2019). This approach involved an inspection of the eigenvalues and variance explained by the components in the residual correlation matrix. To adhere to the Rasch assumption of unidimensionality, the residuals should be random and show no structure (Linacre, 2019). Linacre (2019) suggests that eigen values larger than two are indicative of multidimensionality.

The functioning of the response categories was evaluated by examining category probability curves, frequencies, fit statistics (infit and outfit mean squares), Andrich thresholds and category measures. Infit and outfit statistics exceeding 1.5 are considered problematic (Linacre, 2016). Scale targeting was assessed by analysing the item-person map, where an instrument is considered well-targeted for individuals with average scores on the latent trait if the mean person ability aligns closely with the mean item difficulty around zero. In addition, the difficulty levels of the items were explored to identify their hierarchical ordering.

Items meet Rasch model expectations if their item characteristic curves (ICCs) display the desired empirical structure and fall within the 95% confidence-interval (CI) bands, as well as if their infit and outfit statistics are between 0.75 and 1.30 mean square statistics (Adams & Khoo, 1996; Bond & Fox, 2015; Wilson, 2005). These values were applied to ensure a balance between acceptable model fit and sufficient measurement precision, as well as to align with established best practices (e.g. Wright & Linacre, 1994).

Reliability was investigated using the person and item reliability estimates, and separation indexes. The person reliability estimate and separation index indicate how effectively the scale would replicate the ordering of individuals if the same participants were assessed with a comparable set of items measuring the same construct (Wright & Masters, 1982). A low person separation index (below 2) and person reliability (below 0.80) suggest that the instrument has limited capacity to distinguish between individuals with high and low levels of the underlying trait (Linacre, 2016). The item reliability estimate and separation index reflect the sample’s effectiveness in estimating item difficulty, with higher values indicating that the item hierarchy is likely to remain stable across similar samples (Bond & Fox, 2007; Linacre, 2016).

### Ethical considerations

Ethical approval to conduct this study was obtained from the University of Johannesburg and Department of Industrial Psychology and People Management Research Ethics Committee (No. IPPM-2021-542).

Participants were informed of the anonymous, confidential and voluntary nature of the study. Consent was provided by way of participants clicking a button on the online survey which read ‘I consent to participate in the study’. No incentives were provided for participation.

## Result

The descriptive statistics of the six SU items are shown in Table 1.

**TABLE 1:** Descriptive statistics of the self-undermining scale (*n* = 318).

Items	*M*	s.d.	Med	Skew	Kurt	s.e.
SU1- I make mistakes	2.32	0.77	2	1.58	2.27	0.03
SU2- I admit that I create stress at work	1.67	0.90	1	1.55	2.39	0.04
SU3- I create confusion when I communicate with others at work	1.44	0.74	1	2.03	4.62	0.03
SU4- I create a backlog in my tasks	1.62	0.89	1	1.60	2.33	0.04
SU5- I run into problems at work	1.98	1.05	2	1.16	0.81	0.04
SU6 – I admit that I create conflicts	1.41	0.75	1	2.22	5.52	0.03
SUS	10.43	3.30	10	1.46	2.83	0.14

SU, self-undermining; SUS, self-undermining scale total score; M, mean; Med, median; s.d., standard deviation; s.e., standard error; Skew, skewness; Kurt, Kurtosis.

The unexplained variance in the first, second and third contrast had eigenvalues of 1.49 (11.4%), 1.42 (10.8%) and 1.15 (8.8%), respectively. The eigenvalues below 2 indicate no structure in the residuals, supporting the unidimensionality of the self-undermining scale.

**FIGURE 1:**
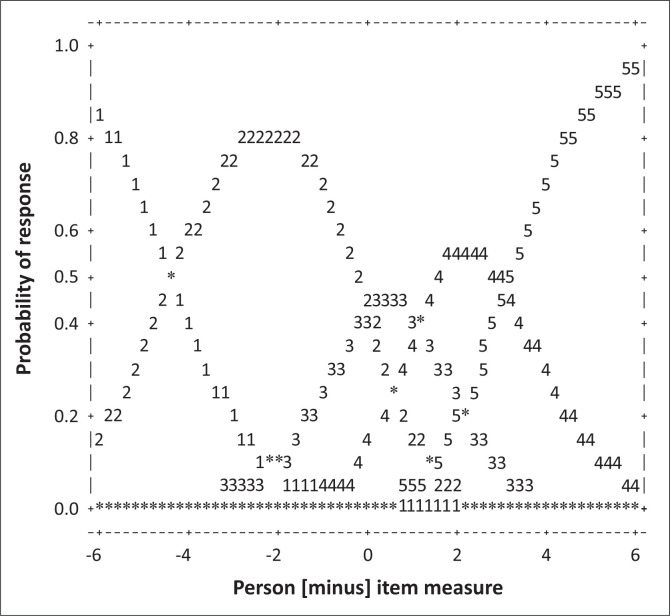
Category probability curves for the self-undermining scale.

Looking at Figure 1, visual inspection of the Rasch-Andrich category probability curves showed that all the five response categories had distinct peaks, suggesting each category measured a distinct part of the latent trait.

Table 2 shows the observed count and percentage of the rating scale categories in the first two columns. For all 318 respondents, categories 1 (*Never*) and 2 (*Sometimes*) were used 34% and 54% of the time, respectively. The rating categories *Regularly* (3), *Often* (4) and *Very Often* (5) captured only 13% of all the responses to the items, suggesting low SU behaviour. With regard to category fit, the infit mean squares ranged from 0.96 to 1.34 and the outfit mean square (MNSQ) ranged from 0.82 to 1.24, indicating generally acceptable fit for each category (Linacre, 2016). Lastly, the Andrich thresholds and category measures advanced across the rating scale, implying that each category in turn was likely to be chosen. Worthy of observation was the large distance between the Andrich thresholds of categories 2 (–4.48 logits) and 3 (0.04 logits), which may correspond to a decline in the statistical information available between these two categories.

**TABLE 2:** A summary of category structure for the self-undermining scale (318 persons, 6 items).

Response category	Observed count	%	Infit MNSQ	Outfit MNSQ	Andrich threshold	Category measure
1 (Never)	648	34	0.96	0.94	-	−5.59
2 (Sometimes)	1016	54	0.98	0.82	−4.48	−2.25
3 (Regularly)	164	9	1.11	1.12	0.04	0.59
4 (Often)	51	3	1.19	1.17	1.27	2.32
5 (Very often)	11	1	1.34	1.24	3.17	4.37

MNSQ, mean square.

Investigation of the SU items began by looking at the summary item and person fit statistics.

The average infit and outfit MNSQ values for persons were 0.97 (s.d. = 0.72) and 0.95 (s.d. = 0.78), respectively. For the items, the average infit and outfit MNSQ values were 1.04 (s.d. = 0.14) and 0.95 (s.d. = 0.18), respectively. Table 3 provides the item measures, standard errors and fit statistics for each item.

**TABLE 3:** Item difficulty for the self-undermining scale.

Item	Measure	s.e.	Infit		Outfit	

			MNSQ	*t*	MNSQ	*t*
SU1	−1.81	0.10	0.86	−1.5	0.74	−2.9
SU2	−0.80	0.11	1.23	2.3	1.18	1.9
SU3	0.92	0.12	0.94	−0.7	0.84	−1.6
SU4	0.53	0.12	1.24	2.7	1.21	2.1
SU5	−0.79	0.11	0.97	−0.3	0.90	−1.1
SU6	1.94	0.14	1.01	0.2	0.85	−1.0
Mean	−3.22	0.85	0.98	−0.10	0.96	−0.05
s.d.	1.25	0.01	0.14	1.6	0.18	1.8

s.e., standard error; MNSQ, mean square; s.d. standard deviation; SU, self-undermining.

The difficulty of the six SU items is located in the column labelled ‘Measure’. Item SU6 (difficulty = 1.94) was the most difficult item to endorse whereas SU1 (difficulty = –1.81) was the easiest to endorse. In terms of item fit, the infit MNSQ ranged from 0.86 to 1.24 and the outfit MNSQ ranged from 0.74 to 1.21. The only potentially misfitting item is SU1 with an outfit MNSQ of 0.74. However, this degree of misfit is minimal and unlikely to have a significant impact on the overall validity or reliability of the measurement model. To inspect the items further, the ICCs were investigated to detect peculiar item behaviour. Item SU1 seemed to show slight misfit in that individuals who were low on SU were scoring higher than expected and those who were high on SU were scoring lower than expected. However, the low frequency count observed for the higher rating scale categories renders this interpretation meaningless.

**FIGURE 2:**
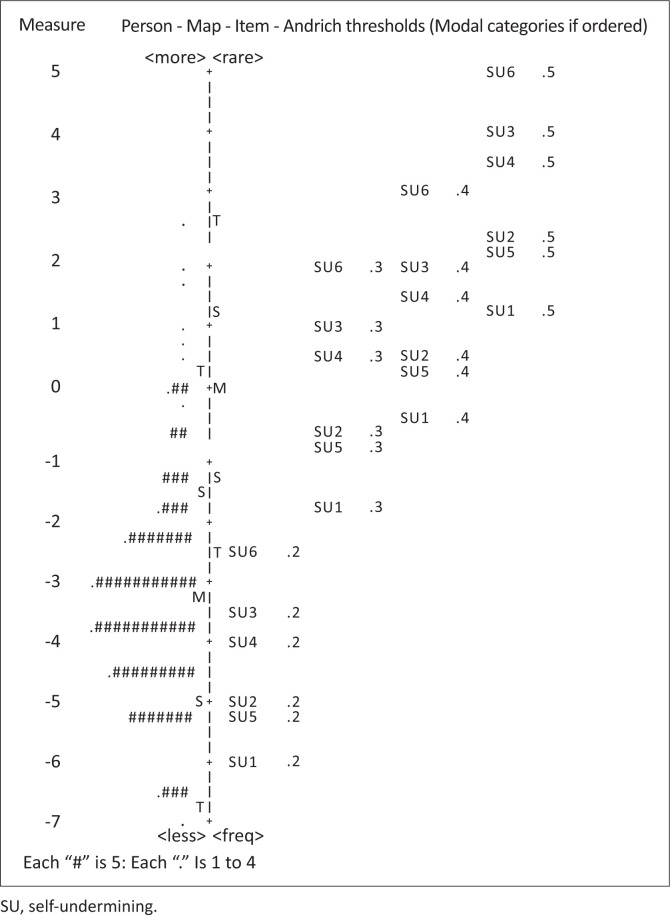
Category thresholds and item/person locations along the self-undermining construct.

Figure 2 displays an item-person map representing all 318 responses to the six SU items. Self-undermining behaviour is marked by the vertical line running across the map which ranged between –7 and 5 logits. The participants are represented by #’s on the left-hand side of the map while the items are represented on the right-hand side of the map. The hierarchical ordering of the items in relation to each category is shown and it can be seen that items SU6 and SU1 were indeed the most difficult and easiest items to endorse, respectively. The map also displays the means (M) for both persons and items. It is shown that the mean item difficulty (0 logits) was greater than the mean person ability (–3 logits), suggesting that the self-undermining scale may have been difficult for this sample to complete or that the items did not measure high levels of SU. Furthermore, the majority of persons were located between –5 and 0 logits which were predominantly measured by rating categories 1 (*Never*) and 2 (*Sometimes*). The three upper-most categories were rarely used among this sample.

The self-undermining scale demonstrated a person reliability of 0.71 and a separation index of 1.57. For the items, the reliability was 0.99, with a separation index of 10.26. The total scale’s internal consistency, measured by Cronbach’s alpha, was 0.77.

## Discussion

### Internal validity and item fit

Bakker and Wang (2020) developed the six-item self-undermining scale to measure the extent to which employees engage in harmful workplace behaviours that create obstacles and undermine their performance at work. In this study, PCA of the Rasch residuals and item fit statistics suggested unidimensional scaling, lending further support to the construct validity of the scale as initially proposed by Bakker and Wang (2020). Satisfactory fit to the Rasch model was observed for all items except for one, SU1 (‘I make mistakes’), which exhibited slight misfit in the form of overfit. While overfit does not severely detract from internal validity, it indicates that SU1 contributes less unique information to the construct than expected (Bond & Fox, 2007).

Misfitting items may compromise the scale’s precision and validity, highlighting the importance of reviewing item content and wording (Bond & Fox, 2007; Linacre, 2002). Item SU1 is particularly open-ended, as it does not specify the context for the mistakes being referenced. This lack of specificity may confuse respondents and lead to varied interpretations of whether the mistakes occur in a work context, home life or other domains. Given that SU is inherently linked to the workplace, reformulating SU1 to read ‘I make mistakes *at work*’ could align it more closely with the intended construct and reduce random responses (Netemeyer et al., 2003). Moreover, the overfit could suggest that SU1 is too general and may not sufficiently differentiate individuals across the SU continuum. Misfit, even when slight, underscores the need for ongoing refinement and evaluation to ensure that each item contributes meaningfully to the overall measurement model (Smith et al., 2008). Addressing the slight misfit of SU1 could enhance the scale’s reliability and precision, particularly in distinguishing between low and high levels of SU behaviour.

### Reliability and rating scale functionality

With regard to the reliability and person-separation, the internal consistency of the self-undermining scale was good, providing support for Bakker and Wang’s (2020) original findings. However, in terms of person-separation, the scale was unable to distinguish between low-versus-high self-underminers in this sample. This was evidenced in the person separation index and the mismatch between the mean item difficulty and the sample’s mean ability depicted on the item-person map. The lack of items measuring average-to-high levels of SU suggests a gap in the scale’s coverage of the construct. The results derived from this sample suggest that the scale is difficult for the average person to endorse. Including items that are easier or more frequent in nature could improve the scale’s ability to differentiate individuals on the SU continuum. This is consistent with best practices in Rasch analysis, which emphasise constructing items that span the full range of the latent trait to enhance measurement precision and targeting (Bond & Fox, 2015).

While the rating scale demonstrated proper ordering in the category thresholds and effectively targeted persons at the lower-end of the SU construct, it did not do as well at targeting those at the higher-end. In Bakker and Wang’s (2020) development study, the majority of participants also endorsed the lowest categories possible (i.e. 1 = Never, 2 = Sometimes), with the three upper-most categories (i.e. 3 = Regularly, 4 = Often, 5 = Very Often) being scarce in their selection. This may be because of the sample not being comfortable in reporting negative work behaviours for fear of punishment (Heneman et al., 1997), their ongoing interest in how they are perceived and evaluated by others (Leary & Kowalski, 1990), or to manage their impressions to avoid blame, maintain their self-esteem and present themselves favourably (Tedeschi & Reiss, 1981). It may also be that the convenient sample used in this study did not include enough individuals with high SU behaviour. This form of sampling bias may be mitigated by purposeful sampling (Smith et al., 2008), and future studies should thus aim to recruit broader samples that include employees who exhibit higher levels of the construct, such as those in high-stress roles (e.g. finance, emergency services) or dysfunctional team environments.

### Limitations

The self-report nature of the research presents the first limitation. It is not uncommon for individuals to practice impression management or faking when completing self-reports on their own behaviour (Tedeschi & Reiss, 1981), especially when the behaviours being monitored are perceived as negative by others. This could be one reason for the low participant scores on SU. The next limitation pertains to the sample. Participants were South African employees and majority identified themselves as White, which is not an accurate representation of the South African population. Caution should thus be exercised in generalising these findings across local and international contexts. Further, the small sample size and its implications for the application of the Rasch Rating Scale Model is a limitation. Larger sample sizes are required for stable parameter estimation in Rasch analyses, particularly when evaluating infrequent category use (Linacre, 1994). Although this study did not use a fully representative sample, it is an initial first step in the right direction. The final limitation concerns the scope of the study which largely focused on the scale’s internal validity and reliability, with little information being available on how SU behaviours, as measured by the scale, predict key workplace outcomes such as job performance, workplace conflict and employee well-being.

### Implications and recommendations

The psychometric properties of the self-undermining scale have not been investigated in South Africa, nor globally, using the Rasch Measurement Model. The study’s findings provide useful insights into the scale’s reliability and internal validity, which may have implications for future researchers and practitioners. For researchers, it may be worth revising the item wording of SU1 to ‘I make mistakes *at work*’ to better capture SU within a working context. Future validation studies should also be undertaken to determine whether alternative rating scales, such as a 4-point Likert scale or behavioural anchors, are better able to differentiate low from high self-underminers. To understand why certain categories were not endorsed and to capture nuances in SU behaviours, qualitative interviews and open-ended questions could also be used. However, because the self-undermining scale effectively captures low-to-moderate levels of SU, practitioners can use it to identify and support employees showing early signs of SU. For local studies conducted in South Africa, cultural differences should be investigated using differential item functioning to explore whether perceptions of SU differ in South Africa compared to other regions, which might influence item responses and rating scale use. Future research should also replicate the study with larger and more diverse samples to determine whether the underutilisation of the higher response categories persist across different populations and contexts. Finally, because the scale measures a non-typical, non-normally distributed construct, researchers should consider ‘other’ reports of SU, such as 360 measures and key performance indicators as opposed to self-reports.

## Conclusion

This study investigated the psychometric properties of the self-undermining scale in a South African context, providing evidence of its unidimensional structure and overall fit to the Rasch model. While the scale effectively targeted individuals with lower levels of SU, it struggled to differentiate higher levels of the construct because of limited item difficulty and infrequent use of upper rating categories. The slight misfit of one item highlights the need for clearer, work-specific wording to enhance construct alignment and reduce test-taker confusion. Future research should focus on refining the scale and validating it in larger, more diverse samples to improve its application across workplace settings.
